# Apparent Skin Discoloration about the Knee Joint: A Rare Sequela of Metallosis after Total Knee Replacement

**DOI:** 10.1155/2015/891904

**Published:** 2015-03-24

**Authors:** Narlaka Jayasekera, Conor Gouk, Amit Patel, Keith Eyres

**Affiliations:** ^1^Royal Devon and Exeter Hospital, Barrack Road, Exeter EX2 50W, UK; ^2^University Hospital North Durham, North Road, Durham DH1 5TW, UK

## Abstract

*Introduction*. Metallosis is a phenomenon most commonly associated with hip replacement. However it can occur in any metallic implant subject to wear. Wear creates metal debris, which is deposited in the surrounding soft tissue. This leads to many local adverse reactions including, but not limited to, implant loosening/osteolysis, pain, and effusion. In the deeper joints, for example, the hip, metal deposits are mostly only seen intraoperatively.* Case Study*. A 74-year-old lady represented to orthopaedic outpatient clinic. Her principle complaint was skin discolouration, associated with pain and swelling over the left knee, on the background of a previous total knee replacement with a metal backed patella resurfacing six years. A plain radiograph revealed loosening of the patellar prosthesis. A diagnosis of metallosis was made; the patient underwent debridement of the stained soft tissue and primary revision of the prosthesis. She remained symptom-free five years after revision.* Discussion*. Metallosis results in metallic debris which causes tissue staining, often hidden within the soft tissue envelope of the hip, but more apparent in the knee. Metallosis may cause pain, effusion, and systemic symptoms because of raised levels of serum-metal ions. Surgical intervention with revision and debridement can have good functional results.

## 1. Introduction

The reaction of metallic debris is that of a foreign-body reaction causing synovitis, as described by Willert and Semlitsch [1]. The body reacts by forming scar tissue and thickened joint capsule through the “formation of granulation tissue, including macrophages and foreign-body giant cells” [1]. This results in restricted range of movement.

The reaction is initially confined to the soft tissues due to the elimination process of the lymphatics, but then this can become overwhelmed and the bone adjacent to the prosthesis can be affected and loosening occurs.

Fluid enriched with the metallic debris causes effusion within the joint. This enriched fluid transports and deposits metal debris about the whole joint leading to discolouration of the soft tissues, inflammation, pain, and effusion.

## 2. Case Study

**Figure 1:**
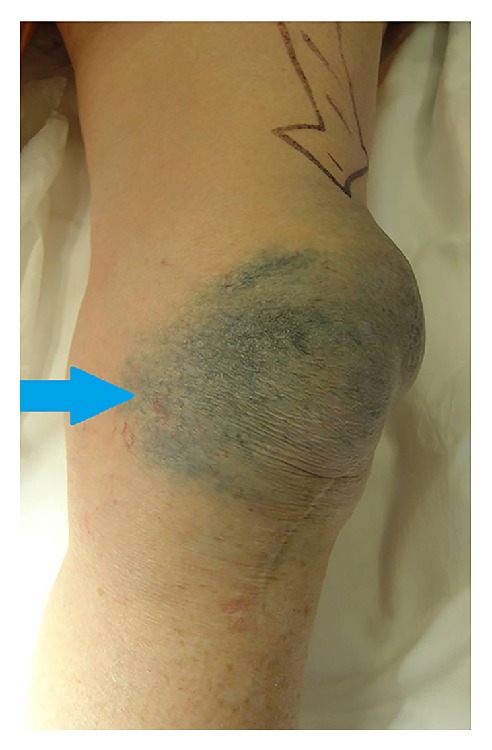
Clinical photograph of the discoloured soft tissue and large effusion secondary to metallosis in the replaced knee.

A 74-year-old woman presented to her GP with a two-year history of increasing skin discolouration, pain, and swelling over the anterior left knee (Figure 1) with an anterior longitudinal midline surgical scar. She had undergone a left total knee replacement six years previously with a metal-backed implant to resurface her patella. Metal-backed patella implants, despite their poor track record [2], are still used to address the difficult scenario of patella deficiency at knee replacement.

**Figure 2:**
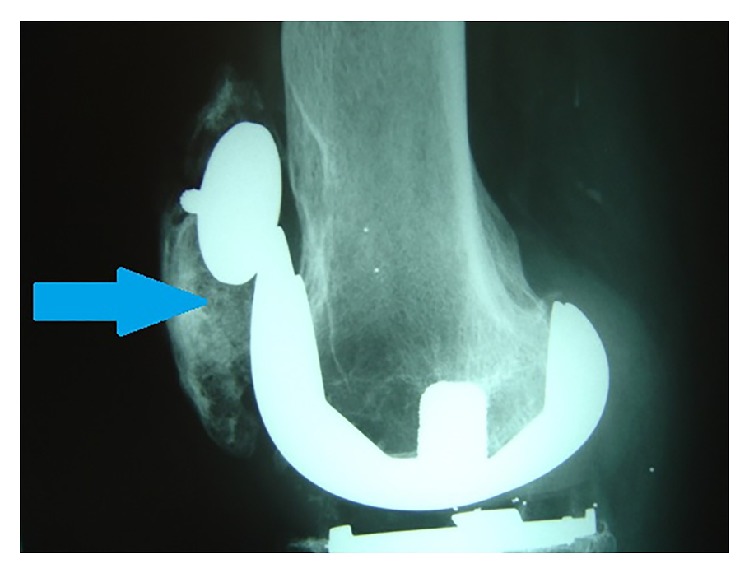
Lateral radiograph of the replaced knee demonstrates the loose patella prosthesis and surrounding osteolysis.

The adverse reaction to metal debris, termed metallosis, may cause loosening/osteolysis of prostheses (Figure 2) secondary to metallic corrosion and release of metal wear debris [3, 4]. Metallosis is characterised by the grey discolouration of the joint tissues, as well as pain, effusion, and elevated serum-metal ion levels. Though commonly implicated with metal on metal hip resurfacing [4, 5], the discolouration and effusion are rarely apparent on clinical inspection because of the considerable soft tissue that envelops the replaced hip joint. However, when metallosis affects the more accessible replaced knee joint, as in this case, the discoloured soft tissue may be obvious on inspection, as would be the large effusion. Systemic effects from raised serum-metal ion levels, particularly cobalt, include neurological deficits: declining sight, hearing, and cognition, cardiac failure/cardiomyopathy, and hypothyroidism.

In this case the patient underwent early revision knee surgery where the stained soft tissues of the knee were debrided and patella component was revised with an alternate bearing surface. The patient remained symptom-free at five-year follow-up.

## 3. Discussion

The orthopaedic community is well aware of the phenomenon of metallosis, although it is most commonly thought of with hip arthroplasty. The hip however has a very substantial soft tissue envelop and is considerably deeper than the knee.

Metallosis should be considered early in the differential diagnoses where discolouration to soft tissues is apparent about a replaced joint. Early debridement and revision should be considered to limit the amount of involved soft tissue. It should be noted that this can often lead to a satisfactory outcome as in this case study.

## 4. Conclusion

Metallosis of a knee implant is important to be documented in literature as it highlights metallosis as a broader phenomenon than just of the hip. It is also of interest as a rare differential diagnosis for advancing pigmentation around a joint, pain, and effusion. The case also demonstrates the importance of knowing the individual implants.
